# Assessment of Vitamin D status in a group of Egyptian children with non alcoholic fatty liver disease (multicenter study)

**DOI:** 10.1186/s12986-016-0112-z

**Published:** 2016-08-19

**Authors:** Amal Mohamed Ahmed, Maha Abdel Ghany, Gehan Lotfy Abdel Hakeem, Aya Kamal, Rania Khattab, Asmaa Abdalla, Laila El Morsi Abou El Fotoh, Abdel Azeem El Mazary, Madiha Abdalla Sayed, Ashraf Mohamed Abdel Fadil

**Affiliations:** 1 Department of Biochemistry, National Hepatology and Tropical Medicine Institute, Cairo, Egypt; 2 Pediatric Department, El Sahel teaching hospital, Cairo, Egypt; 3 Pediatric Department, Minia University, El Minya, Egypt; 4 Public health Department, Faculty of Medicine, Ain Shams University, Cairo, Egypt; 5 Microbiology and Immunology Department, Faculty of Pharmacy, Cairo University, Cairo, Egypt; 6 Department of Clinical Nutrition, Faculty of Applied Medical Science, King Abdul-Aziz University, Jeddah, Kingdom of Saudi Arabia

**Keywords:** Nonalcoholic fatty liver disease, Vitamin D, Children

## Abstract

**Background:**

Nonalcoholic fatty liver disease (NAFLD) is one of the health problems with great burden on the liver that may end with liver cirrhosis and hepatocellular carcinoma. The aim of this work was to assess serum vitamin D level in nonalcoholic fatty liver disease children

**Methods:**

This cross sectional case control study involved 47 patients with nonalcoholic fatty liver disease selected while recruiting the pediatric hepatology clinics. Their ages ranged from 5–15 years and were compared with 23 healthy age and sex matched children. All involved patients were subjected to careful history taking, clinical examination and for patients and control, anthropometric measures for body mass index (BMI) calculation (plotted on WHO percentile growth charts), aspartate aminotransferase (AST), alanine aminotransferase (ALT), alkaline phosphatase (ALP), gamma glutamyl transferase (GGT), bilirubin (total and direct), serum albumin, creatinine, triglycerides, cholesterol, high density lipoprotein (HDL),low density lipoprotein (LDL), fasting blood glucose and fasting insulin (for calculation of insulin resistance), C reactive protein and serum vitamin D all were assayed. NAFLD was detected by ultrasonography and graded as absent, mild, moderate and severe.

**Results:**

Ninety-three percent of NAFLD patients were obese. Significant differences were found between patients and control regarding AST, ALT, ALP, GGT, total and direct bilirubin, serum albumin, creatinine, triglycerides, cholesterol, HDL, fasting blood glucose, fasting insulin, the homeostatic model assessment for insulin resistance (HOMA-IR) and serum vitamin D levels. Significant negative correlation was found between serum vitamin D level and grades of steatosis.

**Conclusions:**

Serum vitamin D level decreases in children with NAFLD. This low serum vitamin D level is associated with higher stages of steatosis but not with BMI.

## Background

Vitamin D is a fat-soluble vitamin formed in the skin from 7-dehydrocholestrol during exposure to solar ultraviolet B (UVB) radiation [1]. 25-hydroxyvitamin D [25(OH)D] is the major circulating metabolite used as indicator of vitamin D stores [2] which is furtherly metabolized in the kidneys to the biologically active form, dihydroxy vitamin D [1,25(OH)_**2**_D]. Vitamin D deficient individuals are more likely to develop alterations in glucose metabolism as impaired glucose tolerance, metabolic syndrome and type 2 diabetes mellitus [3–5]. Based on these evidences, it can be hypothesized that vitamin D deficiency should not be considered an exclusive feature of patients with osteo-mineral disorders. Vitamin D is capable to reduce free fatty acids (FFA) induced insulin resistance both in peripheral tissues and in hepatocytes. Positive associations between 25(OH)D deficiency and the prevalence of obesity have been proved. Recent studies suggested that adequate serum 25(OH)D levels could be associated with increased adipocyte activity and oxidation of fat with the potential of improving of insulin sensitivity which can lead to weight loss [6, 7]. Obese individuals tend to have lower serum vitamin D level than those with normal weights [8, 9]. Several mechanisms have been proposed to explain the low vitamin D levels in obese people and include the sequestration of 25(OH)D by fat tissues as well as body size [10].

Non-alcoholic fatty liver disease (NAFLD) is defined as the presence of steatosis in more than 5 % of hepatocytes in the absence of significant alcohol consumption, drug use or hereditary diseases [11, 12]. NAFLD ranges from simple steatosis, which involves benign fatty infiltration of the liver to a more severe form that involves both steatosis and necroinflammation, known as non-alcoholic steatohepatitis (NASH) which carries increased risks of hepatic fibrosis and cirrhosis [13]. The prevalence of NAFLD had increase over the last two decades which run paralleled the dramatic rise in childhood obesity worldwide [14, 15].

There were no previous studies about serum vitamin D level in the NAFLD children. The aim of this work was to assess the vitamin D status in a group of Egyptian children with nonalcoholic fatty liver disease.

## Methods

Forty-seven patients were selected randomly from those recruited to the pediatric hepatology outpatient clinics in 3 Egyptian centers between Jan 2014 and May 2015 (El Sahel teaching hospital, Minia University children’s hospital and Egyptian national liver institute). Enrolled patients had fulfilled the following criteria: Age (5–15 years) and proved to have NAFLD by ultra-sonographic examination. Candidates were excluded according to the following criteria: Diagnosis of autoimmune liver disease, positive test results for either hepatitis B surface antigen or hepatitis C antibody, history of cardiovascular disease, renal dysfunction, hypo or hyperthyroidism, current use of drugs known to influence 25(OH)D metabolism including glucocorticoids, non-steroidal anti-inflammatory drugs and calcium/vitamin D supplements, severe disability or bone fracture, current infectious condition, presence of tumor, severe anemia and children with type I or II diabetes.

Another 23 healthy age and sex matched children were included as controls selected from healthy school children after exclusion of NAFLD (by ultra-sonographic examination).

All the participants were subjected to detailed clinical history taking and complete physical examination. Measurements of weight (kg) and height (cm) were used to calculate the body mass index (BMI) [=(kg/m^2^)]. BMI readings were plotted on the percentile growth charts for BMI between the age of 2 and 20 years. Childhood obesity was defined as BMI ≥97th percentile while between 3th and 96th centile was considered normal [16].

### Sampling

Five ml of venous blood were collected after an overnight fasting in vacutainers without additive allowed to clot for 30 min at room temperature and centrifuged at 5000 rpm for five minutes. Separated serum was stored into aliquots at- 20 °C until biochemical analysis including liver enzymes (ALT & AST), fasting blood glucose (FBG), total serum cholesterol, triglyceride (TG) and HDL cholesterol (were analyzed enzymatically using kit obtained from Randox Laboratories Limited, Crumlin, UK). Fasting blood glucose and fasting insulin (FINS) was quantified by an electrochemiluminescence immunoassay. Insulin resistance (IR) was assessed by calculating the homeostasis model assessment index for insulin resistance (HOMA-IR) [FBG (mmol/L) × FINS (mU/L)/22.5] [17]. C reactive protein (CRP) concentration was measured using a particle-enhanced immunonephelometry. Serum vitamin D was measured by ELISA (Immunodiagnostic Systems, Louvain-la-Neuve, Belgium).

Liver ultrasound was performed using (GE Healthcare, Logiq 5 pro. ultra sonogram. Real time unit with a 5 MHz abdominal transducer).

Because performing liver biopsies for the exclusive purpose of a study is inappropriate (i.e. for assaying 25(OH)D in NAFLD children), ultra-sonographic diagnosis of NAFLD was made for all study participants according to Saverymuttu et al.,1986 who graded steatosis into four grades: 0 = absent, 1 = mild, 2 = moderate and 3 = severe [18].

The study was carried out according to the principles of the Declaration of Helsinki, and its appendices [19] and was approved by the hospital ethical review board in El Sahel teaching hospital (code 114, August,2015), Minia University hospital (code 75.a, August, 2015) and national liver institute (code 18.c, September, 2015). Written informed consents from the patients^,^ caregivers were obtained for the use of their study-related information and for participation in the ongoing research.

### Statistical analysis

The SPSS (Statistical Package for the Social Sciences) statistical software suite, version 16.0, was used for all statistical analyses (SPSS Inc, Chicago, IL, USA). Data with normal distribution were expressed as the mean values ± SD and were assessed by unpaired Student's *t*-test to evaluate inter-group (NAFLD *vs* control) differences. Comparative analyses of categorical variables were carried out by the chi-square test. The relationship between serum 25(OH)D and demographic and clinical variables were evaluated by partial correlation testing. In addition, regression analysis was performed to identify independent factors of NAFLD. A two-tailed *p* value <0.05 indicated statistical significance.

## Results

Compared to the control group, the NAFLD patients had lower levels of serum 25(OH)D and higher values of BMI centile, fasting blood glucose, serum triglyceride, fasting insulin level, HOMA-IR, ALT, AST and GGT levels (Table 1).

**Table 1:** Some clinical and laboratory data of both studied groups

Parameter		NAFLD patients	Controls	*P*- value
		(No = 47)	(No = 23)	
Age	Mean ± SD	11.13 ± 2.7	10.6 ± 3.1	0.43
Sex	Male (%)	19 (40.4 %)	9 (39.1 %)	0.32
	Female (%)	28 (59.6 %)	14 (60.9 %)	
BMI centile (th)	Mean ± SD	96.8723 ± 0.5	92.2 ± 9.3	<0.001*
ALT (U/L)	Mean ± SD	63.8 ± 31.1	27.6 ± 5.7	<0.001*
AST (U/L)	Mean ± SD	58.8 ± 22.9	28.8 ± 8.7	<0.001*
GGT (U/L)	Mean ± SD	53.8 ± 21.6	34.5 ± 9.4	<0.001*
Serum albumin (mg/dl)	Mean ± SD	3.5 ± 0.52	3.9 ± 0.2	<0.001*
Total bilirubin (mg/dl)	Mean ± SD	1.4 ± 0.71	0.71 ± 0.2	<0.001*
Direct bilirubin (mg/dl)	Mean ± SD	0.32 ± 0.2	0.14 ± 0.05	<0.001*
Alkaline phosphatase (U/L)	Mean ± SD	214.4 ± 56.6	186.2 ± 46.4	0.01*
CRP (mg/dl)	Mean ± SD	56 ± 18.1	5 ± 3.1	<0.001*
Serum creatinine (mg/dl)	Mean ± SD	0.81 ± 0.35	1 ± 0.19	<0.001*
Serum cholesterol (mg/dl)	Mean ± SD	198.3 ± 27.1	163.8 ± 27.5	<0.001*
Serum triglyceride (mg/dl)	Mean ± SD	191.3 ± 37.6	157.7 ± 32.9	<0.001*
Serum LDL (mg/dl)	Mean ± SD	104.7 ± 36.2	108.13 ± 11.1	0.52
Serum HDL (mg/dl)	Mean ± SD	37.3 ± 9.7	43.4 ± 8.1	0.002*
Fasting Blood glucose (mg/dl)	Mean ± SD	143.3 ± 33.9	100.17 ± 14.7	<0.001*
Fasting insulin (mU/ml)	Mean ± SD	11.7 ± 4.8	4.5 ± 2.9	<0.001*
HOMA-IR	Mean ± SD	76.6 ± 41.4	20.7 ± 16.3	<0.001
Serum 25(OH) D (nmol/L)	Mean ± SD	52.1 ± 41.3	104.7 ± 36.2	<0.001*

*Significant (*p* value <0.05), *BMI* body mass index, *ALT* alanine aminotransferase, *AST* aspartate aminotransferase, *GGT* gamma glutamyl transferase, *CRP* C reactive protein, *LDL* Low density lipoprotein, *HDL* High density lipoprotein, *HOMA-IR* homeostasis model assessment index for insulin resistance and 25(OH)*D* 25 hydroxy vitamin D

**Fig. 1:**
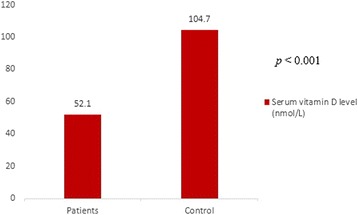
Comparison between studied and control groups regarding serum 25(OH)D

Significant differences were found between patients and controls regarding many laboratory data. Vitamin D level is decreased in patients compared with healthy controls (*p* = 0.001) (Table 2, Fig. 1).

**Fig. 2:**
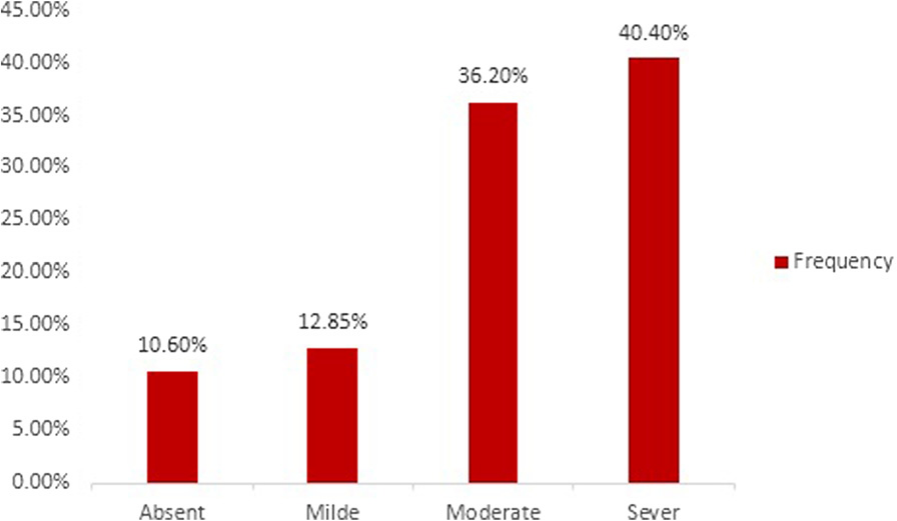
Frequency of different grades of steatosis in NAFLD patients

With stratification of the studied group by grades of steatosis (absent, mild, moderate and severe), five (10.6 %) patients had no steatosis, 6 (12.8 %) had mild steatosis, 17 (36.2 %) had moderate steatosis and 19 (40.4 %) had sever steatosis. Significant differences were found between grades of steatosis regarding serum 25(OH)D except between the moderate and severe group (*p =*1) (Table 2, Fig. 2).

**Table 2:** Comparison between different grades of steatosis regarding serum vitamin D level

Parameter	Grade	Grade	Grade	Grade	Grade	Grade	All
	0 & 1	0&2	0&3	1&2	1&3	2&3	
*p*- value	0.05	<0.001*	<0.001*	<0.001*	<0.001*	1.00	<0.001*

*Significant (*p*-value < 0.05)

**Table 3:** Univariate logistic regression analysis of serum 25(OH)D and hepatic steatosis

Variable	Odd Ratio	Confidence interval (CI)	*p-* value
Serum 25(OH)D	0.888	0.61–1.6	0.53

While vitamin D level is decreased in NAFLD children, it could not be considered as a Dependant factor in these patients (*p* = 0.53) (Table 3).

Forty-nine percent (94 %) of involved patients were obese. Despite of this, there was a non-significant positive correlation between BMI centile and grades of steatosis (*p = 0.94*).

**Fig. 3:**
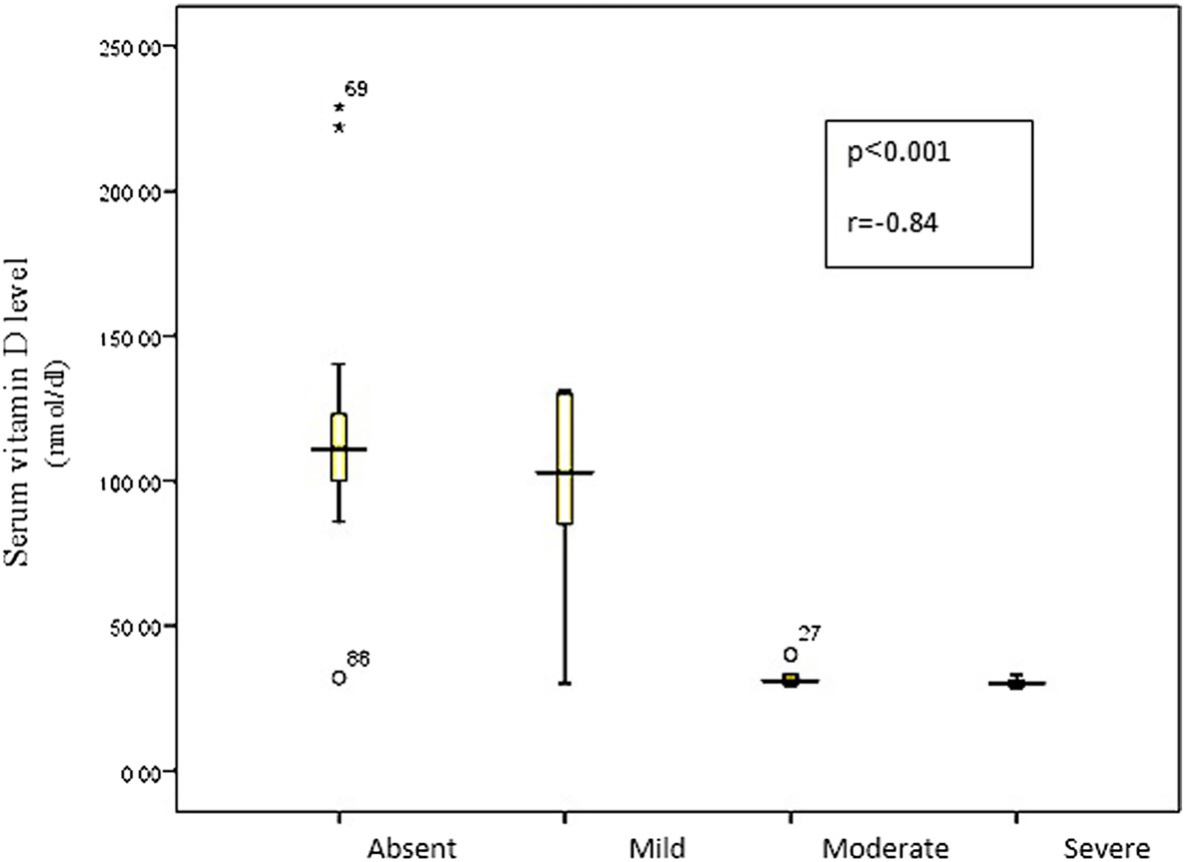
Comparison between different grades of steatosis regarding the level of serum vitamin D

No significant correlation between serum 25(OH)D and age (*p* = 0.11, *r* = −0.24), BMI centile (*p* = 0.6, *r* = −0.8), AST (*p* = 0.83, *r* = 0.03), ALT (*p* = 0.45, *r* = −0.11), GGT (*p* = 0.054, *r* = −0.28), serum albumin (*p* = 0.31, *r* = 0.15), total (*p* = 0.24, *r* = −0.17) and direct bilirubin (*p* = 0.6, *r* = −0.77), serum creatinine (*p* = 0.69, *r* = 0.06), fasting glucose (*p* = 0.16, *r* = −0.21) and fasting insulin levels (*p* = 0.72, *r* = −0.06), serum TG (*p* = 0.41, *r* = 0.12), serum cholesterol (*p* = 0.28, *r* = −0.16), LDL (*p* = 0.46, *r* = 0.11), HDL (*p* = 0.57, *r* = −0.09) and CRP (*p* = 0.66, *r* = −0.7), HOMA-IR (*p* = 0.74, *r* = −0.07). Significant negative correlation was found between serum 25(OH)D and the grades of steatosis (*p* <0.001, *r* = −0.84) (Fig. 3).

Serum 25(OH)D deficiency was not a dependent risk factor for development of childhood nonalcoholic hepatic steatosis as shown by multiple stepwise regression analysis for the grades of steatosis (*p* = 0.54) (Table 3).

## Discussion

To our knowledge, there is no previous study about the level of serum vitamin D in NAFLD in children. In this study, serum 25(OH)D was decreased compared with controls. This comes in agreement with Targher et al., 2007 who performed a liver biopsy to confirm NAFLD. They found significantly lower levels of serum 25(OH)D in NAFLD patients than in controls [20].

Obesity was found in most NAFLD children but no significant correlation was detected between serum 25(OH)D and BMI. Excess body fat results in its increased sequestration and low availability and, as a consequence, low serum 25(OH) D levels [10, 21]. Further study suggested the dilution of vitamin D in body tissue mass (in fat cell mass as well as extracellular fluid) rather than sequestration in the fat tissue [22].

Vitamin D dose response in obese subjects is about 30 % lower than the response in non-obese ones. In addition, the dose–response curves in obese and non-obese subjects are effectively parallel.

Lagunova et al. 2009 found that obese patients have about 20 % lower levels of serum 25(OH)D compared with normal weight or overweight persons [23].

Low serum 25(OH)D level might play a role in NAFLD pathogenesis, possibly via suppression of its anti-inflammatory properties. Indeed, chronic inflammation is considered a key factor in NAFLD progression [24, 25]. Increased visceral adiposity promotes the release of both free fatty acids and pro-inflammatory cytokines [26]. In turn, activation of inflammation within the liver, possibly via increased nuclear factor k B (NFkB) activity, thus promoting downstream transcription of pro-inflammatory cytokines and ultimately perpetuating a vicious cycle [27]. Increased levels of serum 25(OH)D might act to reduce the development/progression of NAFLD by countering these inflammatory processes. Lower serum 25(OH)D levels are associated with higher circulating inflammatory markers [28–31].

Inadequate 25(OH)D status in healthy individuals is associated with increases in both vascular endothelial cell NFkB protein expression and vascular NFkB signaling Hence, low serum 25(OH)D levels may promote these inflammatory processes that are also involved in the development and progression of NAFLD [32].

A different study opposes our results supporting that CYP27A1 and CYP2R1 enzyme expression, as well as the ability to hydroxylize vitamin D_3_, are well persevered in NASH and do not contribute to lowered serum 25(OH)D levels [33].

We found no significant correlation between BMI and serum 25(OH)D despite of obesity in a large number of patient. This is against Saneei et al., 2013 who proved significant inverse weak correlation between serum 25(OH)D levels and BMI in adult populations [34].

In the present study there was inverse negative correlation between vitamin D and the grades of steatosis. Jablonski et al., 2013 proved a strong inverse relationship between NAFLD and 25(OH)D levels [35]. Vitamin D directly regulates the metabolism of FFAs via its action on peroxisome proliferator-activated receptor gamma (PPAR-γ) [36]. Studies were made to prove weather fatty liver disease could be a consequence of increased inflammation, or does inflammation increase hepatic steatosis. An association between hepatic steatosis and elevation of CRP, a protein produced predominately by the liver under conditions of inflammation found by Ndumele and colleagues [37]. Hepatic steatosis may exaggerate the synthesis of high sensitivity CRP or other mediators by the liver, thereby increasing its systemic levels. Other laboratory studies have shown that inflammation is not a consequence of steatosis, but could be its cause [38, 39]. Thus, in patients with NAFLD, the inflammatory response likely functions as an amplification loop contributing to hepatic steatosis.

Rhee et al., 2013 found a minor but significant difference in 25(OH)D levels between patients with and without NAFLD [40].

## Conclusions

Serum vitamin D level decreases in children with NAFLD. This low serum vitamin D is associated with higher stages of steatosis but not with BMI.

### Limitations of the study

1. The small number of patients included in the study owing to the rarity of hepatic steatosis in children.
2. Liver biopsy could not be performed to either patients or controls for accurate diagnosis of NAFLD considering the ethics of humanity.

## Abbreviations

25 (OH)D, twenty five hydroxyl vitamin D; 25(OH)_2_ D,twenty five di hydroxyl vitamin D; ALP, alkaline phosphatase; ALT, alanine aminotransferase; AST, aspartate aminotransferase; BMI, body mass index; CRP, C reactive protein; ELISA, enzyme-linked immunosorbent assay; FFAs, free fatty acids; FINS, fasting insulin; FPG, fasting blood glucose; GGT, gamma glutamyl transferase; HDL, High density lipoprotein; HOMA-IR, homeostatic model assessment of insulin resistance; IL-6, interleukin-6; IR, insulin resistance; LDL, low density lipoprotein; NAFLD, nonalcoholic fatty liver disease; NASH, nonalcoholic steatohepatitis; PPAR-γ, peroxisome proliferator-activated receptor gamma; TG, triglycerides; UVB, ultraviolet ray B; VDR, vitamin D resistance; WHO, World Health Organization
