# Erratum to: Assessment of Vitamin D status in a group of Egyptian children with non alcoholic fatty liver disease (multicenter study)

**DOI:** 10.1186/s12986-016-0140-8

**Published:** 2016-11-18

**Authors:** Amal Ahmed Mohamed, Maha Abdel Ghany, Gehan Lotfy Abdel Hakeem, Aya Mostafa, Rania Abdelmonem Khattab, Asmaa Mahmoud Abdalla, Laila El Morsi Abou El Fotoh, Abdel Azeem El Mazary, Madiha Abdalla Sayed, Ashraf Mohamed Abdel Fadil

**Affiliations:** 1 Department of Biochemistry, National Hepatology and Tropical Medicine Institute, Cairo, Egypt; 2 Pediatric Department, El Sahel teaching hospital, Cairo, Egypt; 3 Pediatric Department, Minia University, El Minya, Egypt; 4 Department of Community, Environmental, and Occupational Medicine, Faculty of Medicine, Ain Shams University, Cairo, Egypt; 5 Microbiology and Immunology Department, Faculty of Pharmacy, Cairo University, Cairo, Egypt; 6 Department of Clinical Nutrition, Faculty of Applied Medical Science, King Abdul-Aziz University, Jeddah, Kingdom of Saudi Arabia

## Erratum

After publication of this article [1], the authors noticed errors to the author list, Authors’ Contributions section and the author affiliation for Aya Mostafa.

Amal Ahmed Mohamed was incorrectly refered to as Amal Mohamed Ahmed; Aya Mostafa was incorrectly referred to as Aya Kamal; Rania Abdelmonem Khattab was incorrectly referred to as Rania Khattab; and, Asmaa Mahmoud Abdalla was incorrectly referred to as Asmaa Abdalla. The correct author list is included in this erratum.

Following this, it was noticed the Authors’ Contributions section was incorrect. The correct Authors’ Contributions section is included in this erratum.

In addition to this, Aya Mostafa’s affiliation was incorrectly included in the original article [1]. The correct affiliation information is included in the Author details section of this erratum.
